# Emotion regulation and self-inhibition’s association with mental health outcomes, caregiver strain, and well-being in parents of autistic children: a dyadic analysis

**DOI:** 10.1186/s11689-026-09704-1

**Published:** 2026-05-08

**Authors:** Leonardo Dominguez Ortega, Alexandra Sturm, Meghan M. Krushena, Amanda C. Gulsrud

**Affiliations:** 1 Semel Institute for Neuroscience and Human Behavior, University of California, Los Angeles, 760 Westwood Plaza, Los Angeles, CA 90024 USA; 2 Department of Psychology, University of Southern California, 3620 McClintock Ave SGM 501, Los Angeles, CA 90089 USA; 3 Department of Psychological Science, Loyola Marymount University, 1 Loyola Marymount University Dr., Los Angeles, CA 90045 USA; 4 College of Medicine, Central Michigan University, 1200 S. Franklin St., Mount Pleasant, Michigan 48859 USA

**Keywords:** Parents of autistic children, Actor-partner interdependence model, Emotion regulation, Self-inhibition, Mental health, Caregiver strain, Well-being

## Abstract

**Background:**

Parents of autistic children report more depression, anxiety, and caregiver strain, and poorer well-being than parents of non-autistic children. Though more research has begun to investigate how parent-specific factors may influence these outcomes, few consider cognitive factors like emotion regulation or self-inhibition. These skills may be particularly relevant given their documented benefits and associations with mental health and well-being in the general population. An important consideration when investigating the needs of parents is the interconnectedness of the family unit. Thus, methodologies that consider this shared context are essential to appropriately support resilience of parents of autistic children.

**Methods:**

Our sample consisted of 263 different-sex parent dyads with at least one child formally diagnosed with autism spectrum disorder. Using the Actor-Partner Interdependence Model, we assessed the links between parents’ emotion regulation and self-inhibition and their own and their partner’s depression and anxiety symptoms, caregiver strain, and well-being. Interdependence was established using correlations given the distinguishable nature of our dyads.

**Results:**

Both mothers’ and fathers’ emotion regulation were associated with their own depression and anxiety symptoms, caregiver strain, and well-being. Stronger emotion regulation was associated with fewer mental health symptoms, less caregiver strain, and better well-being. There was only one significant association for self-inhibition: stronger self-inhibition scores in fathers were linked to better well-being. We did not observe any associations between parents’ emotion regulation or self-inhibition and partner outcomes after false discovery rate correction.

**Conclusions:**

Emotion regulation, and not self-inhibition, emerged as an important source of resilience for mental health and well-being of parents of autistic children. Previous work has looked to reduce caregiver strain in this population through interventions that directly and indirectly target emotion regulation. Our findings highlight the need for further research on interventions that both directly target parents’ emotion regulation skills and modify environmental contexts (e.g., social support, respite care) to enhance regulatory capacity and support parental resilience. As our work was cross-sectional, future work should investigate the causal relationship between emotion regulation, mental health, and well-being in parents of autistic children.

## Background

It is well established that parents of autistic children are more likely to report poorer well-being, more negative mental health outcomes (e.g., depression and anxiety), and higher caregiver strain compared to parents of non-autistic children. [1–6] Research points to the importance of core cognitive processes, namely inhibition and emotion regulation, in the management of mental health, stress, and well-being among the general population. [7, 8] In autism research, however, parent cognitive traits are often used as predictors of child outcomes rather than examining intraindividual processes that may promote parent well-being. [6, 9] Identifying factors that bolster parent’s resilience to adverse parental mental health outcomes and that strengthen well-being is critical. [10, 11] Additionally, most of the work targeting parents focuses on a single parent within a household without considering the potential support of a co-parent’s cognitive resources. [12, 13] The present study aims to understand the associations between parent cognition (i.e. self-inhibition, emotion regulation) and parent mental health, well-being and caregiver strain within parent dyads of autistic children.

### Emotion regulation and self-inhibition

Emotion regulation and self-inhibition are aspects of cognition that promote positive mental health and well-being outcomes. [9] Among individuals who experience higher levels of stress relative to the general population, they are especially critical. [14] This extends to parents of autistic children who must navigate behavioral challenges, [3, 4] complex medical and educational systems, [15] and social stigma. [16] Parent training studies where parents are taught strategies to manage child characteristics (e.g. behavior management) do not systematically reduce parent stress and strain. [17] In contrast, interventions targeting the psychological health and emotion regulation of parents of autistic children have demonstrated efficacy. [18].

Emotion regulation includes the processes and strategies that individuals use to manage, influence, and respond to their emotional experiences. [6, 19] Studies in the general population have found that emotion regulation can aid depression and anxiety symptom management, [1, 20–24] promote well-being, [25] and reduce caregiver strain. [26] Additional work in parents of autistic children has further highlighted the connection between emotion regulation and well-being. For example, parents of autistic children who report using reappraisal, an emotion regulation-based coping strategy, had greater well-being. [16] Further, a systematic review by Curley et al. (2025) [10] identified increased parental resilience as a key contributor to well-being among parents of autistic children. Many of the resilience themes highlighted in the review utilize emotion regulation (i.e. cognitive reappraisal), including “catching the silver lining,” “adopting a mindset of acceptance,” “realizing the value of optimism,” and “embracing the positives in life.” However, no work has simultaneously examined in both mothers and fathers how emotion regulation facilitates positive mental health and well-being and reduces strain among parents of autistic children.

While emotion regulation focuses on a parents ability to manage their own emotions, self-inhibition focuses on control over one’s own impulses and emotional urges to act. [19] Like emotion regulation, self-inhibition is also related to one’s own mental health. It has been associated with better well-being [27] and better response to depression treatment in the general population. [28] Conversely, difficulties with inhibitory control can play a critical role in blunting one’s ability to manage stress. [29] However, the relative contribution of self-inhibition to the mental health and well-being of parents of autistic children has not yet been investigated. Researchers have called for investigation of the etiology of adverse stress and mental health among parents of autistic children. [17] Thus, a deeper exploration of emotion regulation, self-inhibition, and their relationship to psychopathological symptoms, well-being, and caregiver strain in parents of autistic children is needed.

### Including fathers in dyadic models of parenting

An important limitation of this area of work is that parent characteristics, often a mother’s, are examined at the individual level. Although often missing from studies in general, fathers are particularly understudied in autism research. [30] One systematic review found that mothers outnumbered fathers in research participation by a factor of eight, [13] despite fathers’ significant involvement in their children’s lives. [31] Theories to explain this disparity include fathers being inaccessible (e.g., employment restrictions, lack of time) and more difficult to recruit than mothers, [32] fathers not being asked to participate, [33] and mothers often fulfilling the role of primary caregiver. [34] Because primary caregivers are often relied upon to provide critical information relevant to pediatric research (e.g., daily habits, behaviors), fathers’ contributions have been limited. [35] This leaves an important gap in the literature as research has found that parenting dyads are highly interconnected. [36] At a basic level, a shared child will present a similar influence over a parenting dyad. Within these circumstances, parents may work together to manage shared parenting demands. For example, it has been found that partners can serve as a primary source of support for one another when addressing parenting needs. [12] This work is further supported by research that outlines the bidirectional relationships between parents’ physical and mental health. [37] Thus, the inclusion of fathers in research focused on parent characteristics will not only highlight fathers’ unique contributions, but will also provide a more complete understanding of the parenting dyad.

The Actor-Partner Interdependence Model (APIM) [38] is a statistical model that considers interdependence by assessing the way parents influence themselves (actor effects) and the way they influence their partner (partner effects). A benefit of this statistical model is its standardization of calculating interdependence amongst paired observations through correlations and its flexibility in fitting data from distinguishable (e.g., different-sex parent dyads) and indistinguishable dyads (e.g., same-sex parent dyads). [38] This model also considers dyad members shared context by controlling for partners’ predictor scores when estimating actor effects and tests for additional factors that could increase model fit by correlating residuals from each dyad member. [38].

### Aims & current study

The current study sought to investigate the associations between parents’ emotion regulation and self-inhibition and their own and their partner’s anxiety and depression symptoms, caregiver strain, and well-being using the Actor-Partner Interdependence Model. [38] This study leveraged a large, United States-based sample of parent dyads with at least one biological autistic child. It fills important gaps in existing literature by including fathers, assessing the role of parent cognitive characteristics on their mental health outcomes, and by considering the interdependent nature of the parenting dyad. We hypothesized that stronger parent emotion regulation and self-inhibition would be associated with lower anxiety and depression symptoms, lower caregiver strain, and higher well-being (actor effects). Additionally, we hypothesized that strong parent emotion regulation and self-inhibition would be associated with less anxiety and depression symptoms, less caregiver strain, and higher well-being in their partners (partner effects).

## Methods

### SPARK and Research Match

Simons Powering Autism Research for Knowledge (SPARK) is an ongoing nationwide study funded by the Simons Foundation Autism Research Initiative (SFARI). It collects survey data and biospecimens from autistic individuals of any age and their immediate, biological family members. The study’s goal is to create a large data repository with the intention of improving the field’s understanding of autism and to serve the community through their direct study involvement and participation service development. Individuals with a professional diagnosis of autism (e.g., diagnosed by a medical professional, clinical psychologist) who reside in the United States are eligible for study participation. Registration, collection of biospecimens, and surveys can be completed remotely or at one of 30 + affiliated clinical sites across the country. Registration consists of creating a profile with basic demographic and diagnostic information, along with providing consent (parent/guardian for minors and conserved adults, or self for independent adults) for the phenotypic and genetic portions of the study separately. Additional surveys regarding behavior and family medical information can be completed upon registration or at later dates, with select surveys available at regular intervals (e.g., yearly). See Feliciano et al. [39, 40] for additional SPARK protocol information.

Research Match, a service of SFARI, allows approved researchers to contact subsets of the SPARK cohort with additional volunteer research opportunities to supplement standard SPARK data collection. The current study utilized data collected from a subset of the SPARK cohort upon approval from the SPARK Participant Access Committee (PAC). The SPARK PAC is comprised of SPARK staff, independent researchers, and community representatives who review applications on a quarterly basis. A battery of online surveys was provided to qualified participants (defined below) who accepted a study invitation and consented to participation.

### Participants

At the time of data collection, SPARK had enrolled 106,577 autistic participants of all ages. Our Research Match sample included *N* = 263 autistic children along with both biological parents (*N* = 263 parent-child triads; *N* = 789 individuals). The current study utilized data from parent dyads (*N* = 263 different-sex parent dyads, *N* = 526 individuals). Inclusion criteria included participation from both biological parents, though cohabitation or legal partnership was not required. All autistic participants were required to be under the age of 18 at the time of Research Match data collection (January through February 2021).

### Measures

#### Demographic questionnaire

Information on child biological sex, diagnosis (official diagnosis, age of diagnosis), and language level were collected during participants’ initial SPARK registration prior to Research Match participation. Our study-specific survey (i.e., collected during the Research Match data collection process) included an additional demographic questionnaire that queried parents’ age, autism diagnostic status (self-identifying, professionally diagnosed, or no diagnosis), marital status (married, widowed, divorced, separated, never married, living with partner), and the number of children under the age of 18 years in the household.

#### Barkley Deficits in Executive Functioning Scale

The Barkley Deficits in Executive Functioning Scale (EF; BDEFS), [41] completed by parents as part of our Research Match-specific battery, is a self-report questionnaire of adult EF abilities that contains five subscales: self-management of time, self-organization/problem-solving, self-restraint, self-motivation, and self-regulation of emotions (*N* = 89 items). Items from the self-restraint (also referred to as self-inhibition; *n =* 19 items) and self-regulation of emotions (also referred to as emotion regulation; *n =* 13 items) subscales were culled for the current study. The self-inhibition subscale aims to measure participants’ impulsivity and ability to inhibit responses. The emotion regulation subscale focuses on respondents’ ability to manage emotions. All items were answered on a 4-point Likert scale (1 = *“Never or rarely”* to 4 = *“Very often”*). The BDEFS had excellent internal consistency in the current sample (self-inhibition, α = 0.93; emotion regulation, α = 0.92), replicating psychometrics from prior studies (α = 0.92 for the entire measure). [41] An item sum was calculated for each subscale to create a subscale score, with higher scores indicating increased deficits. Possible scores for self-inhibition and emotion regulation ranged from 19 to 76 and 13–52, respectively.

#### Generalized Anxiety Disorder-7

The Generalized Anxiety Disorder-7 (GAD-7), [42] collected for the present Research Match study, was used to assess the presence and severity of anxiety symptoms in parents. Participants were asked to report how often they were bothered by anxiety symptoms in the past two weeks using 7 items rated on a 4-point Likert scale (0 = *“Not at all”* to 3 = *“Nearly every day”*). The GAD-7 has been found to have strong psychometric properties, with excellent internal consistency in the current sample (α = 0.91), similar to prior studies (Cronbach α = 0.92), [42] and good test-retest reliability in the reference sample (ICC = 0.83). [42] Additionally, this measure has been found to have strong evidence of criterion, construct, and factorial validity. [50] The GAD-7 yields a total sum score with possible scores ranging from 0 to 21 with higher scores indicating greater levels of anxiety.

#### Patient Health Questionnaire-9

The Patient Health Questionnaire-9 (PHQ-9) [43] was used to assess depression symptoms in parents and was part of our Research Match-specific survey battery. Each of the nine items represents an established criterion for major depression as listed in the DSM-IV. The PHQ-9 is a tool that can provide a depressive disorder diagnosis and grade the severity of depression symptoms (e.g., minor to severe symptom presentation) in the context of the previous two weeks. Individuals responded to each item on a 4-point Likert scale (0 = *“Not at all”* to 3 = *“Nearly every day”*). The PHQ-9 was found to have good internal consistency in the current sample (α = 0.89), similar to prior studies (α = 0.86–0.89; test-retest reliability in the reference sample, 0.84). [51] It has also been found to have strong construct and criterion validity. [43] We removed item nine, which asks about suicidality, due to a lack of active clinical monitoring of survey data and upon recommendation from the PAC. Thus, our measure yielded a total sum score that ranged from 0 to 24 using eight items that represent the severity of respondents’ depression symptoms. Higher scores indicated more depression symptoms. Given the exclusion of item nine, raw scores should be interpreted with caution and should not be compared to studies that include the full scale.

#### Caregiver Strain Questionnaire–Short Form

Internalized caregiver strain from the previous six months was assessed using the Caregiver Strain Questionnaire-Short Form (CGSQ-SF), [44] an abbreviated version of the Caregiver Strain Questionnaire. [45] It consists of 10 items that query objective strain (*n* = 6 items) and subjective internalized strain (*n* = 4 items). This survey was part of our Research Match battery. Responses were measured using a 5-point Likert scale (1 = *“Not at all”* to 5 = *“Very much a problem”*). Items were prefaced with “In the past 6 months, how much of a problem was the following…” The internal consistency of the CGSQ-SF is comparable to that of the original form (α = 0.90),^44^ which was replicated in the current sample (α = 0.91). Total scores were calculated by summing items, with higher scores indicating higher caregiver strain. Possible scores ranged from 10 to 50.

#### Wellbeing Scale

The Wellbeing Scale is a modified version of Ryff’s Scales of Psychological Well Being [46, 47] and was used to assess parent well-being during Research Match data collection efforts. This self-report questionnaire consists of 18 items assessing six aspects of well-being: self-acceptance, autonomy, environmental mastery, purpose in life, positive relations with others, and personal growth. Responses were measured on a 7-point Likert scale (1 = *“Strongly agree”* to 7 = *“Strongly disagree”*) and item scores were averaged to create a total score. The Wellbeing Scale demonstrated good internal consistency in the current sample (α = 0.86), similar to prior studies. [48] Possible scores ranged from one to seven with higher scores representing greater well-being.

#### Child characteristics

Child characteristics, namely child age (months), biological sex (male, female), and language level were collected during initial registration for the SPARK study and were included as covariates in our models. For language level, parents were asked to describe their autistic child’s language abilities using the following options: “No words/does not speak,” “Uses single words meaningfully (for example, to request),” “Combines 3 words together into short sentences,” and “Uses longer sentences of his/her own and is able to tell you something that happened.”

### Analytic plan

RStudio version 4.2.3 [49] was used to calculate descriptive statistics, bivariate raw correlations, and mean comparisons (see Tables 1, 2 and 3). The Actor-Partner Interdependence Model [38] guided our main analyses interrogating the impact of parents’ emotion regulation and self-inhibition on their own and their partner’s depression and anxiety symptoms, caregiver strain, and well-being. As specified by Cook & Kenny, [38] our first step was to establish interdependence between mothers’ and fathers’ corresponding variables (e.g., mothers’ and fathers’ well-being). Given our dyads were distinguishable (i.e., there is a meaningful difference between dyad members – their biological sex) we employed a series of two-tailed Pearson’s raw correlations. We also specified a liberal alpha of 0.20 as highlighted in Cook & Kenny’s [38] work where a statistically significant finding would indicate interdependence.

Subsequently, APIM models were conducted using path analysis in Mplus software. [50] We assessed the links between parents’ emotion regulation and self-inhibition on their own and their partner’s anxiety and depression symptoms, caregiver strain, and well-being. Four models were run in total: all used parents’ emotion regulation and self-inhibition as predictors and either depression, anxiety, caregiver strain, or well-being as the outcome variables. Child age, biological sex, and language level were included as covariates, predicting mother’s and father’s outcomes. Given the large number of estimated parameters (56 in total), we accounted for false discovery rate (FDR) using the Benjamini-Hochberg procedure. The proportion of false positives was set to *q* = 0.05. Results below represent standardized coefficients and FDR corrected *p*-values.

## Results

### Preliminary analyses

Mothers in our sample were, on average, 40.6 years old (*SD* = 7.01) and fathers were, on average, 42.9 years old (*SD* = 7.74). Most parents reported being married (481; 91.44%), 19 reported living with a partner (3.61%), 15 were divorced (2.85%), six were never married (1.14%), four reported being separated from their partner, and one parent was widowed. As mentioned before, parent dyads were not required to cohabitate or be legally married, meaning their reported marital status may reflect their relationship with a partner who is not the other biological parent of their autistic child registered in SPARK. For child demographic information, see Table 1.

**Table 1:** Child Demographic Characteristics

	Female	Male	Total
Counts	56	195	251
Age at Registration (Years)	7.59 (3.82)	7.95 (3.94)	7.87 (3.91)
Language Level			
Longer Sentences	34	100	134
Combines 3 Words	7	37	44
Uses Single Words	5	31	36
No Words	9	27	36
Missing	1	0	1
Cognitive Impairment	7	15	22
Race			
Asian	3	8	11
Black or African American	3	7	10
Native American	0	2	2
Native Hawaiian or Pacific Islander	1	0	1
White	27	126	153
Other	1	6	7
Missing	21	46	67
Ethnicity			
Hispanic	4	24	28
Not Hispanic	31	125	156
Missing	21	46	67

Child demographics were collected during SPARK registration, not during Research Match data collection. Data were missing for 12 children

Table 2 presents descriptive statistics for parents’ self-inhibition, emotion regulation, caregiver strain, anxiety, depression, and well-being by parent sex. Using established cutoffs for the PHQ-9, 98 mothers (37.3%) and 120 fathers (45.6%) reported low levels of depression symptom severity (or lacked it all together; scores: 0–4), 72 mothers (27.4%) and 70 fathers (26.6%) reported being in the mild range (scores: 5–9), 47 mothers (17.9%) and 34 fathers (12.9%) reported being in the moderate range (scores: 10–14), 35 mothers (13.3%) and 27 fathers (10.3%) reported being in the moderately severe range (scores: 15–19), and 8 mothers and fathers each (3%) reported severe levels of depression symptoms (scores: 20–24). Data were missing for three mothers and four fathers. For the GAD-7, 75 mothers (28.5%) and 103 fathers (39.2%) reported minimal anxiety severity (scores: 0–4), 97 mothers (36.9%) and 92 fathers (35%) reported mild severity (scores: 5–9), 45 mothers (17.1%) and 37 fathers (14.1%) reported moderate anxiety severity (scores: 10–14), and 37 mothers (17.1%) and 19 fathers (10.3%) provided scores within the severe range (scores: >14). Data were missing for one mother and four fathers. More than half of mothers and fathers reported depression and anxiety symptom levels within the minimal to mild range; about a third or less were within the moderate to severe ranges.

**Table 2:** Parent Means and SDs for Variables of Interest

	Self-Inhibition (BDEFS)	Emotion Regulation(BDEFS)	GAD-7	PHQ-9	CGSQ-SF	Wellbeing Scale
	M (SD)	M (SD)	M (SD)	M (SD)	M (SD)	M (SD)
Mothers	30.25(9.83)	22.98(8.31)	8.08(5.70)	7.64(5.86)	28.08(9.76)	5.10(0.84)
Fathers	33.75(9.53)	23.32(8.00)	6.61(5.23)	6.73(6.00)	26.28(9.20)	5.02(0.88)

PHQ-9 scores have a truncated range of 0–24 as item 9 was excluded. Raw scores should not be compared to other studies that use the full scale

A series of paired sample t-tests and Wilcoxon signed rank tests (for variables with non-normally distributed difference scores between mothers and fathers) found that mothers reported significantly more caregiver strain (*d* = 0.182, *p =.*004), depression symptoms (*d* = 0.11, *p =.*03) and anxiety symptoms (*d* = 0.217, *p* <.001). Conversely, fathers reported higher self-inhibition difficulties (*d* = 0.294, *p* <.001). Parents did not differ in self-reported well-being or emotion regulation (see Table 2). These results held after Benjamini-Hochberg procedure corrections to account for FDR. The *p*-values of our preliminary correlations to establish interdependence fell below our alpha of 0.20, indicating interdependence amongst mothers’ and fathers’ outcomes and can be found in Table 3.

**Table 3:** Correlation Matrix of Outcome Variables (Tests of Interdependence)

	Mothers’ Anxiety	Mothers’ Depression	Mothers’ Caregiver Strain	Mothers’Well-being
Fathers’Anxiety	**r** = **0.12**, **p** = .05*	r = 0.14 p < 0.001*	r = 0.06 p =. 19*	r = −0.11 p =. 02*
Fathers’ Depression	r = 0.15 p =. 01*	**r** = **0.24**, **p** < 0.001*	r = 0.10 p =. 06*	r = −0.12 p =. 02*
Fathers’ Caregiver Strain	r = 0.17 p < 0.01*	r = 0.18 p < 0.01*	**r** = **0.54**, **p** < 0.001*	r = −0.12 p =.13*
Fathers’Well-being	r = −0.11 p =.03*	r = −0.20 p < 0.01*	r = −0.11 p =.02*	**r** = **0.22**, **p** < 0.001*

Bolded values represent the analyses used to determine interdependence amongst mother and father scores. *p <.20

### Depression

#### Actor effects

The model linking parents’ emotion regulation and self-inhibition skills to their depression symptoms found that higher emotion regulation difficulties in mothers were associated with more depression symptoms (*ß* = 0.460, *p* <.007). Additionally, higher emotion regulation difficulties in fathers were associated with more depression symptoms (*ß* = 0.521, *p* <.007). Neither mothers’ (*ß* = 0.108, *p =.*485) nor fathers’ (*ß* = 0.090, *p =.*528) self-inhibition skills were associated with their own symptoms.

#### Partner effects

This same model revealed no significant partner effects. Neither mothers’ emotion regulation or self-inhibition skills (mothers’ emotion regulation linked to fathers’ depression: *ß* = 0.063, *p =.*710; mothers’ self-inhibition linked to fathers’ depression: *ß* = 0.025, *p =.*849) nor fathers’ emotion regulation or self-inhibition skills (fathers’ emotion regulation linked to mothers’ depression: *ß* = −0.091, *p =.*495; fathers’ self-inhibition linked to mothers’ depression: *ß* = 0.142, *p =.*268) were associated with their partner’s depression symptoms.

**Fig. 1:**
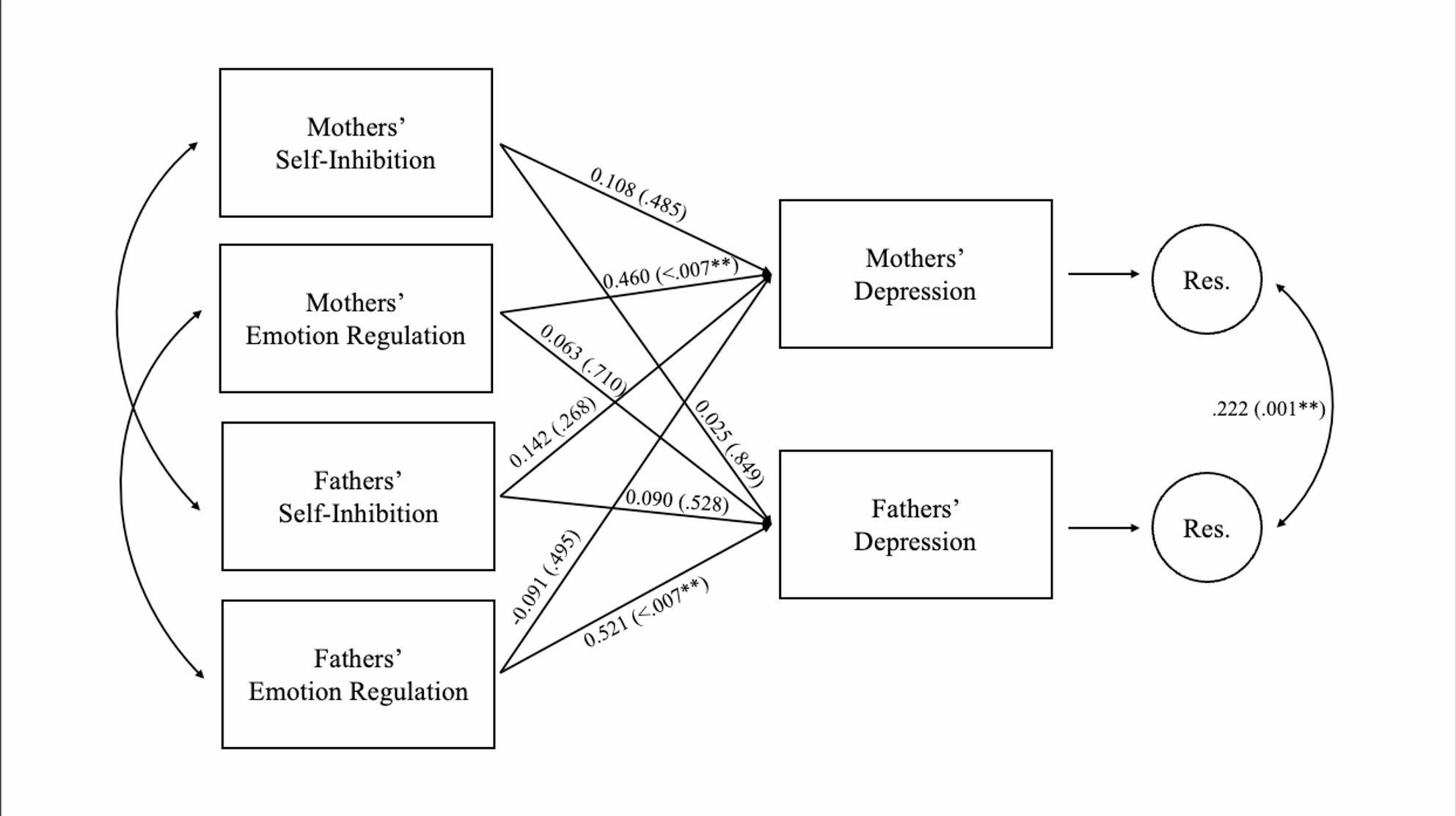
Parents’ Depression APIM Results Note. Presented coefficients are standardized. FDR corrected p-values are located within parentheses. Covariates were included in the model but are not included in this figure for simplicity. Res. = Residual. * <.05, ** <.01, *** <.001

Child age, biological sex, and language level were not associated with mothers’ (*ß* = −0.046, *p* =.679; *ß* = 0.015, *p* =.849; *ß* = 0.018, *p* =.849, respectively) nor fathers’ depression symptoms (*ß* = −0.021, *p* =.849; *ß* = 0.060, *p* =.528; *ß* = 0.005, *p* =.952, respectively). The residual correlation between parents’ symptoms was statistically significant (*ß* = 0.222, *p* =.001), suggesting the potential benefit of including additional predictors that may explain shared variability between mother’s and father’s depression symptoms. Figure 1 maps the results of this analysis to a corresponding path diagram.

### Anxiety

#### Actor effects

The model linking parents’ emotion regulation and self-inhibition skills to their anxiety symptoms found that higher emotion regulation difficulties in mothers were associated with more anxiety symptoms (*ß* = 0.457, *p* <.007). Additionally, higher emotion regulation difficulties in fathers were also associated with more anxiety symptoms (*ß* = 0.608, *p* <.007). In other words, parents with increased difficulties with emotion regulation skills self-reported more symptoms. However, neither fathers’ (*ß* = 0.020 *p =.*847) nor mothers’ (*ß* = 0.180, *p =.*237) self-inhibition were associated with their own anxiety symptoms.

Partner effects

This same model revealed no significant partner effects. Neither mothers’ emotion regulation or self-inhibition skills (mothers’ emotion regulation linked to fathers’ anxiety: *ß* = 0.063, *p =.*710; mothers’ self-inhibition linked to fathers’ anxiety: *ß* = 0.063, *p =.*715) nor fathers’ emotion regulation or self-inhibition skills (fathers’ emotion regulation’s linked to mothers’ anxiety: *ß* = −0.135, *p =.*205; fathers’ self-inhibition’s linked to mothers’ anxiety: *ß* = 0.109, *p =.*379) were associated with their partner’s anxiety.

**Fig. 2:**
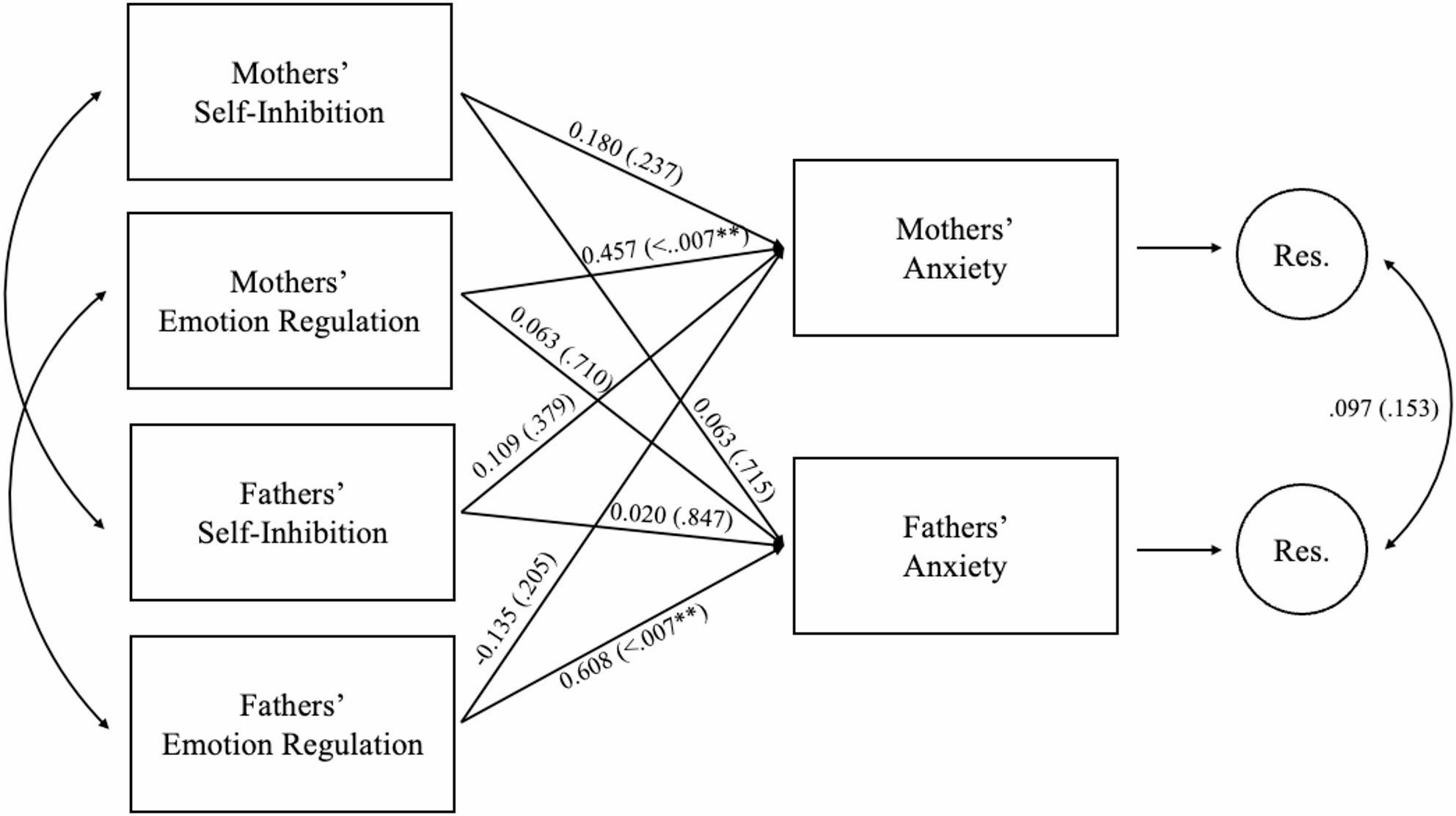
Parents’ Anxiety APIM Results Note. Presented coefficients are standardized. FDR corrected p-values are located within parentheses. Covariates were included in the model but are not included in this figure for simplicity. Res. = Residual. * <.05, ** <.01, *** <.001

Child language level was significantly associated with fathers’ anxiety symptoms such that higher child language abilities were linked to more anxiety symptoms (*ß* = 0.142, *p* =.028). Child language level was not associated with mothers’ anxiety symptoms (*ß* = 0.082, *p* =.360). Child age and biological sex were not associated with mothers’ anxiety symptoms (*ß* = −0.085, *p* =.347; *ß* = 0.058, *p* =.495, respectively) nor fathers’ anxiety symptoms (*ß* = −0.045, *p* =.668; *ß* = 0.062, *p* =.424, respectively). The residual correlation between parents’ symptoms was not significant (*ß* = 0.097, *p =.*153), suggesting our model accounted for a significant portion of the shared variance between parents’ anxiety. Figure 2 maps the results of this analysis to a corresponding path diagram.

### Caregiver strain

#### Actor effects

The model linking parents’ emotion regulation and self-inhibition skills to caregiver strain found that higher emotion regulation difficulties in mothers were associated with higher levels of own caregiver strain (*ß* = 0.274, *p* <.007). Additionally, higher emotion regulation difficulties in fathers were associated with higher levels of their own caregiver strain (*ß* = 0.280, *p* =.012). Neither mothers’ (*ß* = 0.035, *p =.*849) nor fathers’ (*ß* = −0.034, *p =.*849) self-inhibition was associated with their own strain.

#### Partner effects

This same model found no significant partner effects. Neither mothers’ emotion regulation or self-inhibition skills (mothers’ emotion regulation linked to father’s caregiver strain: *ß* = 0.074, *p =.*715; mothers’ self-inhibition linked to fathers’ caregiver strain: *ß* = 0.115, *p =.*528) nor fathers’ emotion regulation or self-inhibition skills (fathers’ emotion regulation linked to mothers’ caregiver strain: *ß* = −0.025, *p =.*847; fathers’ self-inhibition linked to mothers’ caregiver strain: *ß* = −0.025, *p =.*847) were associated with their partner’s caregiver strain.

**Fig. 3:**
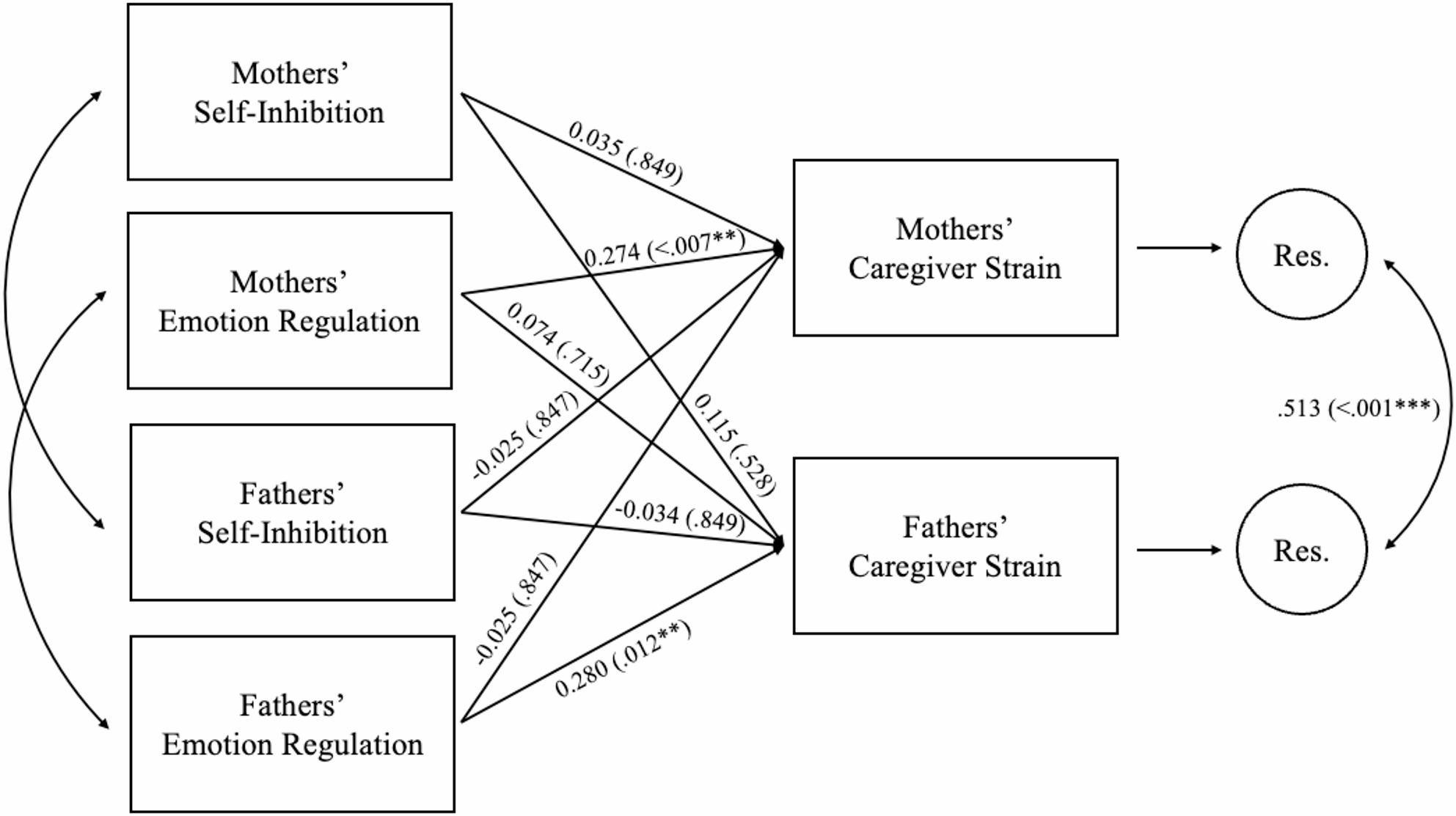
Parents’ Caregiver Strain APIM Results Note. Presented coefficients are standardized. FDR corrected p-values are located within parentheses. Covariates were included in the model but are not included in this figure for simplicity. Res. = Residual. * <.05, ** <.01, *** <.001

Child age, biological sex, and language level were not associated with mothers’ caregiver strain (*ß* = −0.022, *p* =.849; *ß* = 0.123, *p* =.146; *ß* = 0.129, *p* =.136, respectively) nor fathers’ caregiver strain (*ß* = 0.005, *p* =.952; *ß* = 0.062, *p* =.528; *ß* = 0.106, *p* =.237, respectively). The residual correlation between parents’ strain was statistically significant (*ß* = 0.513, *p* <.001), suggesting the potential benefit of including additional predictors that may explain shared variability between mother’s and father’s caregiver strain. Figure 3 maps the results of this analysis to a corresponding path diagram.

### Well-being

#### Actor effects

The model linking parents’ emotion regulation and self-inhibition skills to well-being found that higher emotion regulation difficulties in mothers were associated with lower levels of their own well-being (*ß* = −0.473, *p* <.007). Additionally, higher emotion regulation difficulties in fathers were associated with lower levels of their own well-being (*ß* = −0.288, *p* =.007). Mothers’ self-inhibition was not associated with their own well-being (*ß* = −0.058, *p =.*710), but fathers’ self-inhibition *was* linked to their own well-being (*ß* = −0.318, *p* <.007) such that fathers’ difficulties with self-inhibition were associated with poor well-being.

#### Partner effects

This same model found no significant partner effects. Neither mothers’ emotion regulation or self-inhibition skills (mothers’ emotion regulation linked to father’s well-being: *ß* = −0.178, *p =.*146; mothers’ self-inhibition linked to fathers’ well-being: *ß* = 0.043, *p =.*815) nor fathers’ emotion regulation or self-inhibition skills (fathers’ emotion regulation linked to mothers’ well-being: *ß* = 0.068, *p =.*710; fathers’ self-inhibition linked to mothers’ well-being: : *ß* = −0.146, *p =.*258) were associated with their partner’s well-being.

**Fig. 4:**
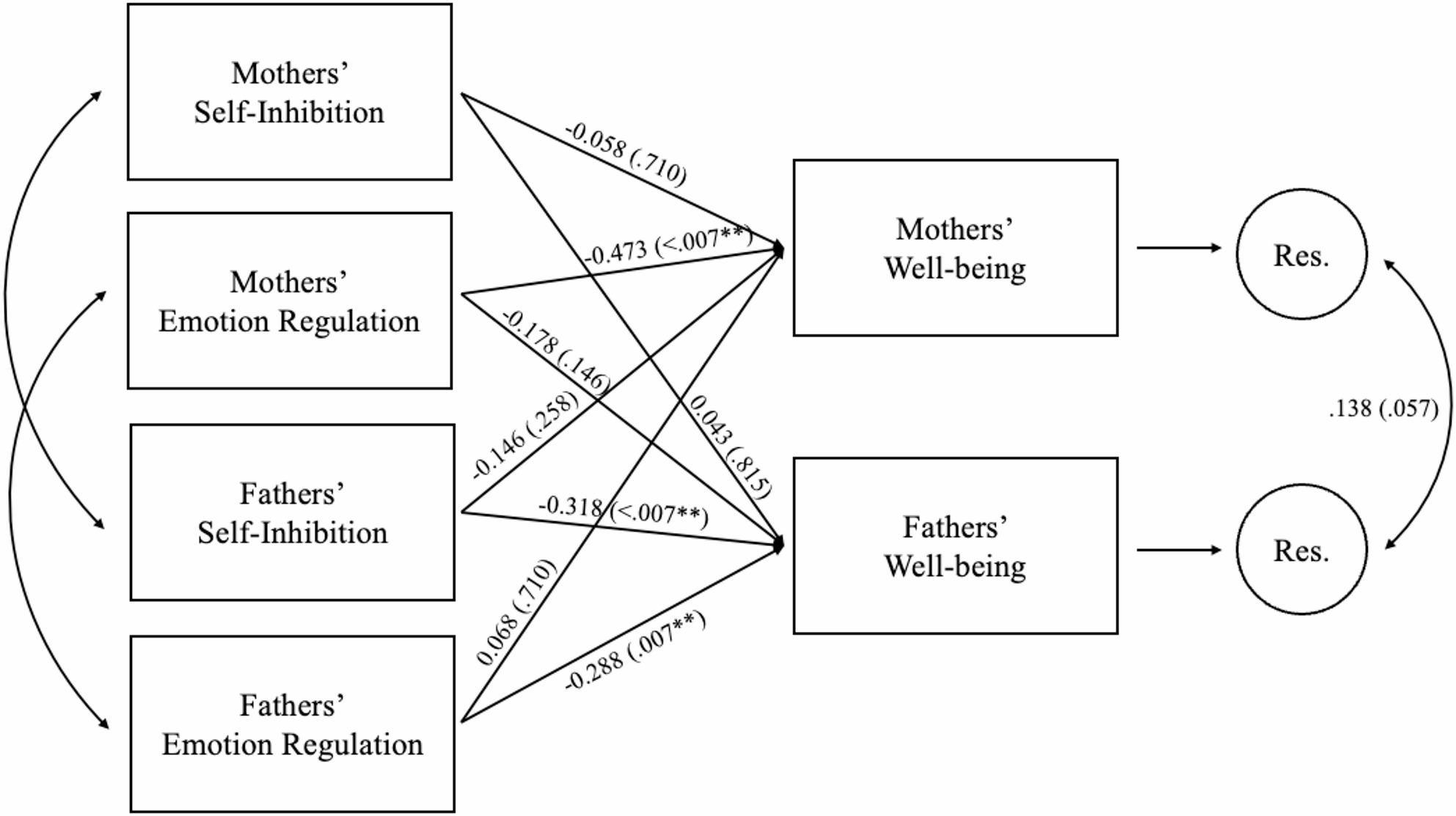
Parents’ Well-Being APIM Results Note. Presented coefficients are standardized. FDR corrected p-values are located within parentheses. Covariates were included in the model but are not included in this figure for simplicity. Res. = Residual. * <.05, ** <.01, *** <.001

Child age, biological sex, and language level were not associated with mothers’ caregiver strain (*ß* = 0.022, *p* =.849; *ß* = −0.103, *p* =.200; *ß* = −0.029, *p* =.849, respectively) nor fathers’ caregiver strain (*ß* = 0.050, *p* =.679; *ß* = 0.005, *p* =.952; *ß* = 0.001, *p* =.988, respectively). The residual correlation between parents’ well-being was not statistically significant (*ß* = 0.068, *p =.*053) suggesting our model accounted for a significant portion of the shared variance between parents’ well-being. Figure 4 maps the results of this analysis to a corresponding path diagram.

## Discussion

The present study sought to investigate the association between parents’ emotion regulation and self-inhibition and their own and their partner’s depression and anxiety symptoms, caregiver strain, and well-being. We used the Actor-Partner Interdependence Model to leverage a large, U.S.-based sample of 263 parent dyads with an autistic child. This study yielded several important findings. Consistent with our prediction and research in the general population, parents’ emotion regulation was associated with parents’ own mental health outcomes (depression, anxiety), caregiver strain, and well-being. [20, 22–25, 51] In contrast, self-inhibition was only associated with fathers’ well-being. Neither parent’s emotion regulation nor self-inhibition predicted the other parent’s mental health, caregiver strain, or well-being. Further, child language level was significantly associated with fathers’ anxiety such that increased language abilities in children were linked to more anxiety symptoms.

Findings from the present study suggest that emotion regulation is a critical source of resilience for both mothers and fathers of autistic children. Emotion regulation was significantly associated with each of the indicators of psychological health, namely depression, anxiety, caregiver strain, and well-being. Though the present study examined emotion regulation broadly, findings in the general population have identified an association between specific emotion regulatory processes and symptoms of depression and anxiety that should be explored in future work within autistic parents. For example, in the general population and in an undergraduate sample, use of maladaptive emotion regulation strategies has been associated with depression symptoms and anxiety symptoms. [19, 51, 52] Prior research in parents of autistic children shows that adaptive coping strategies involving emotion regulation (i.e., cognitive reappraisal) can buffer the negative effects of stress. [6–27] These regulatory strategies also contribute positively to parental well-being. [10] While research has previously investigated the effects of parent training to support child cognition (e.g. executive function) on caregiver strain in parents of autistic children, [53, 54] our work highlights the importance of directly supporting parent emotion regulation.

Though relationship between emotion regulation and mental health, caregiver strain and well-being were universal for both parents, self-inhibition was uniquely related to father’s sense of well-being. The experiences of fathers of autistic children are qualitatively distinct from mothers in several ways, including gendered familial roles, [55] differences in their emotional reaction to their child’s diagnosis [56] and management of ongoing child-related stressors, and engagement with different coping strategies. [57] It is possible that some of the demands specific to fathers may place a greater resource demand on inhibitory skills. For example, fathers report feeling more anger than do mothers, stemming from a feeling of helplessness when they are unable to manage their child’s behavior. [56] In the general population, strength of anger following anger induction is related to poor inhibitory control. [58] Future research will need to carefully consider how self-inhibition uniquely impacts fathers’ well-being, particularly as this may be an important intervention target to promote paternal resilience.

In the present study, we identified no significant impact of an individual’s cognition (emotion regulation and self-inhibition) on their partner’s psychological well-being. Nonetheless, significant correlations between parent’s anxiety, depression, well-being, and caregiver strain, respectively, suggest interdependence between mother and father outcomes. Further, we identified significant residual shared variance for nearly all dyad’s mental health outcomes that was unaccounted for by emotion regulation and self-inhibition. Significant bivariate correlations and significant residual correlations accounting for self-inhibition and emotion regulation suggest that there are family-specific factors that influence both mother’s and father’s psychological well-being that should be explored. Though the child-specific factors included in the present analyses (age, biological sex, language level) largely did not impact parent psychological well-being, other child-related traits (e.g. behavior problems) identified by parents as stress-inducing should also be explored.

Findings from the present study have several important implications. Results could aid the development of direct and indirect supports aimed at improving parent emotion regulation, subsequently supporting resilience to adverse mental health outcomes. Studies have demonstrated efficacy in reducing caregiver stress and strain by using approaches that integrate emotion regulation directly (i.e. cognitive/coping strategies) and indirectly (e.g. relaxation, mindfulness, stress reduction). [17] Additionally, future work should also focus on modifiable aspects of the environment (e.g. social support, respite) that may increase regulatory resources for parents.

The current study is not without limitations. Importantly, though our sample consists of parents of autistic children, we examined only the influence of three child-level traits (age, biological sex, language level) on parents’ depression or anxiety symptoms, caregiver strain, or well-being. Future work should consider additional child-level factors, including externalizing behaviors. The severity of behavioral symptoms or parents perceptions of those behaviors may be stronger predictors of parent psychological well-being. [1, 6] Though relevant to ASD, child age, biological sex, and language level do not test direct links between ASD and parent outcomes. Additionally, our study did not exclusively include cohabitating couples, which could provide better insight into the interdependence of our variables of interest. This may have impacted the lack of partner effects observed in our study given less overlap and shared parenting experiences. Lastly, our study did not explicitly investigate the mechanisms behind emotion regulation’s impact on parent outcomes. Future research is needed that interrogates the directionality of these effects to better understand the impact of emotion regulation on mental health, caregiver strain, and well-being in parents of autistic children.

In conclusion, the present study supports the importance of emotion regulation in parents of autistic children and identifies emotion regulation as a key predictor of both mothers’ and fathers’ psychological well-being. This study also found that the influence of emotion regulation on psychological well-being is largely an intraindividual process. Given the significance of the relationship between dyad’s psychological well-being outcomes after controlling for self-inhibition and emotion regulation, there are as-yet unidentified constructs that would inform how parent outcomes are interdependent. Our inclusion of fathers and use of dyad-level analyses in this sample is also of note. Given fathers are understudied [30] and research often includes the perspective of a sole caregiver (usually mothers),^34^ these findings fill an important gap by incorporating fathers’ experiences and providing a more complete picture of parenting. Future work in this area would benefit from the inclusion of child behavior symptoms in explanatory models, the inclusion of additional family-level factors that may impact these analysis, and longitudinal studies that could determine the directionality and mechanisms behind these effects.

## Data Availability

Data used in this study are publicly available through the Research Match arm of the Simons Powering Autism Research for Knowledge (SPARK) Study. For further details, please contact the corresponding author.

## Abbreviations

APIM: Actor-Partner Interdependence Model
SPARK: Simons Powering Autism Research for Knowledge
SFARI: Simons Foundation Autism Research Initiative
PAC: Participant Access Committee
EF: Executive function
BDEFS: Barkley Deficits in Executive Functioning Scale
GAD-7: Generalized Anxiety Disorder-7
PHQ-9: Patient Health Questionnaire-9
CGSQ-SF: Caregiver Strain Questionnaire-Short Form
FDR: False discovery rate
